# A New Role for Browning as a Redox and Stress Adaptive Mechanism?

**DOI:** 10.3389/fendo.2015.00158

**Published:** 2015-10-09

**Authors:** Yannick Jeanson, Audrey Carrière, Louis Casteilla

**Affiliations:** ^1^UMR STROMALab, CNRS 5273, INSERM U1031, Université Toulouse III – Paul Sabatier, Toulouse, France

**Keywords:** adipose tissue plasticity, adipocytes, redox, uncoupling protein-1, browning, stress

## Abstract

The worldwide epidemic of obesity and metabolic disorders is focusing the attention of the scientific community on white adipose tissue (WAT) and its biology. This tissue is characterized not only by its capability to change in size and shape but also by its heterogeneity and versatility. WAT can be converted into brown fat-like tissue according to different physiological and pathophysiological situations. The expression of uncoupling protein-1 in brown-like adipocytes changes their function from energy storage to energy dissipation. This plasticity, named browning, was recently rediscovered and convergent recent accounts, including in humans, have revived the idea of using these oxidative cells to fight against metabolic diseases. Furthermore, recent reports suggest that, beside the increased energy dissipation and thermogenesis that may have adverse effects in situations such as cancer-associated cachexia and massive burns, browning could be also considered as an adaptive stress response to high redox pressure and to major stress that could help to maintain tissue homeostasis and integrity. The aim of this review is to summarize the current knowledge concerning brown adipocytes and the browning process and also to explore unexpected putative role(s) for these cells. While it is important to find new browning inducers to limit energy stores and metabolic diseases, it also appears crucial to develop new browning inhibitors to limit adverse energy dissipation in wasting-associated syndromes.

In the past, brown and white adipocytes were described as classically located in brown and white fat, respectively. Although these cells shared the name of adipocytes, their functions were traditionally opposed and those of brown adipocytes were strongly associated with the thermoregulation process and energy dissipation. The first discoveries that brown adipocytes could be detected in white fat were made in the 1980–1990s and revealed an unexpected plasticity of white adipose tissue (WAT), largely driven by β3-agonist, at least in rodents (1–3) and dogs (4). Unfortunately, the lack of efficiency of β3-agonist treatment in humans caused interest in this field to wane. However, in recent years, the rediscovery of uncoupling protein-1 (UCP1)-expressing cells in white fat together with the simultaneous demonstration of the presence of functional brown adipocytes in human adults have been accompanied by very new insights into the developmental origins of the different types of adipocytes. These ground breaking discoveries have completely changed our view on adipose tissue plasticity and generated great enthusiasm associated with a huge number of publications in the field, reporting unexpected results. The present review tries to summarize these recent findings and discusses new perspectives concerning unexpected roles and functions of UCP1-expressing cells.

## White Adipocytes, Brown Adipocytes, and Adipose Tissue Plasticity: A Historical Perspective

### White Adipocytes

Mature white adipocytes play a fundamental role in energy homeostasis and constitute the main stores of energy in the form of triglycerides (5). They display variable size (25–200 μm) and a unilocular morphology. In addition to their metabolic functions, white adipocytes have strong endocrine activity and secrete a variety of molecules commonly called adipokines, highlighting the fact that adipose tissue has interactions with other organs in the body (6). These cells are mainly localized in WAT, which are found at distinct anatomical sites. Subcutaneous and visceral fat pads (mesenteric, perigonadal, perirenal, etc.) are classically distinguished because the subcutaneous WAT has a protective action whereas the visceral fat pad is thought to be more deleterious in the development of obesity and related metabolic diseases (type II diabetes, cardiovascular disease, etc.) (7–9).

### Brown Adipocytes and UCP1 Expression

Brown adipose tissue (BAT) contains brown adipocytes, which are smaller (15–60 μm) than white adipocytes and have a multilocular morphology (5). Brown adipocytes are highly oxidative cells that release energy as heat (10, 11) thanks to their high mitochondrial content and to the expression of UCP1. This protein, inserted in the inner mitochondrial membrane, acts as a proton channel and uncouples the production of ATP by ATP synthase from respiratory chain functioning. This leads to the activation of catabolism and subsequent heat production. This non-shivering thermogenesis plays a key role in body temperature homeostasis (10, 11) as definitively demonstrated by UCP1-deficient mice, which are cold sensitive (12). Moreover, the presence of large amounts of BAT in species like sheep or hibernates, which require active thermogenesis to protect the new-born animals from cold exposure on a snow-covered pasture in spring or to emerge from hibernation, also argues that BAT evolved primarily to function as a thermogenic system to maintain body temperature (11, 13). BAT is more vascularized than WAT and is probably among the most vascularized tissues of the body (14). Such vascularization is required for the integrity of BAT. It was recently demonstrated that reducing the vasculature specifically in adipose tissues induced the whitening of BAT (15). In a temperate environment, UCP1 is inhibited by di- and triphosphate nucleotides. During cold exposure, UCP1 is strongly and quickly activated by the sympathetic nervous system (SNS) (16). The local release of noradrenaline in BAT activates lipolysis, which induces the release of free fatty acids. These constitute not only oxidative substrates for brown adipocytes but also potent activators of UCP1 (17–19). The same adrenergic stimulus up-regulates the expression of UCP1 at the transcriptional level via activation of the PKA-p38 mitogen-activated protein kinase signaling pathway that induces the phosphorylation and activation of molecular transactivators of UCP1 (19, 20). With increasing time of cold exposure, SNS triggers a trophic response through activation of mitochondriogenesis that also contributes to the increase of the global thermogenic capacity of brown adipocytes (21). Recently, noradrenaline has also been shown to induce the phosphorylation of dynamin-related protein-1, which induces the fragmentation of brown adipocyte mitochondria. This fragmentation promotes uncoupling and mitochondrial depolarization (22). Lastly, in addition to the activation of the thermogenic program of mature brown adipocytes, noradrenaline also induces the proliferation of brown progenitors through the stimulation of β1-adrenergic receptors and their differentiation (23).

In addition to their role in maintaining body temperature, brown fat cells possess strong oxidative potential and can thus also burn excessive calories (11, 24–26). Transplantation of BAT into the visceral cavity of mice fed with a high-fat diet or having the ob/ob genetic background increases glucose tolerance and improves insulin sensitivity (26, 27). However, UCP1-deficient mice are not obese when kept at room temperature (21°C) (12). The onset of obesity is only observed when these mice are housed at thermal neutrality, exempt from any thermal stress (28). These findings underscore the importance of thermogenesis in the energy balance of homeothermic animals and can explain the different relative importance of diet-induced thermogenesis related to BAT activity according to the developmental status or the species (29). Because of their small size, rodents and many new-born mammals are permanently in thermogenic deficit, which leads to low grade but permanent BAT activation. Despite these limitations, when brown fat cells are stimulated, they exhibit properties that offer hope of new therapies as the cells constitute putative targets in the fight against metabolic diseases such as diabetes and obesity (30).

The beneficial effects of brown adipocytes on energy metabolism are also due to their endocrine/paracrine activity (31). It has long been known that brown adipocytes release triiodothyronine from thyroxine by their deiodase activity, thus stimulating their thermogenic capacity (32). More recently, the secretion of fibroblast growth factor-21 (FGF21), besides vascular endothelial growth factor, fibroblast growth factor-2, interleukin-6, and neural growth factor (31, 33–35), has been attracting much attention. High levels of FGF21, a protein originally known to be produced by the liver in response to fasting, are produced and secreted into the bloodstream by BAT, especially during thermogenic activation (36). FGF21 acts on many tissues, including WAT, the liver, pancreas, heart, or central nervous system (31). Furthermore, some adipokines, such as leptin, adiponectin, and resistin, formerly recognized as specifically secreted by white adipocytes (37, 38), are also secreted by brown adipocytes. By analogy with the term “adipokines” for proteins secreted by white adipocytes, molecules secreted by brown adipocytes have sometimes been referred to as “batokines” (26, 31).

Until the discovery of a third type of adipocyte, things were very simple and focused on UCP1 only: the absence or presence of UCP1 in an adipocyte defined the white or brown phenotype, respectively. Thus, many studies investigated the control of UCP1 gene expression and first focused on catecholamines and thiiodothyronine pathways (19, 39). In addition to cAMP-dependent responsive element binding protein and thyroid hormone receptor, other molecules, including CCAAT/enhancer binding protein-β transcription factor, retinoic acid receptor, activating transcription factor-2, and peroxisome proliferator-activated receptors gamma and alpha (PPARγ and PPARα), strongly up-regulate the expression of UCP1 at transcriptional level (40, 41). Among all these regulatory proteins, PPARγ plays a pivotal role and mice having non-functional PPARγ exhibit brown adipocytes with a unilocular lipid droplet (looking like white adipocytes!) and a decrease in UCP1 expression after cold exposure (42). The presence of PPARγ agonists, such as the anti-diabetic agent rosiglitazone, is required for the expression of UCP1 in adipocytes differentiated in primary culture (43). Various co-regulators, such as peroxisome proliferator-activated receptor coactivator-1α (PGC-1α) and the positive regulatory domain containing-16 protein (PRDM16) are also crucial. The expression of PGC-1α is regulated by the PKA pathway (44) and, *in vitro* as well as *in vivo*, overexpression of PGC-1α induces the expression of UCP1 along with other mitochondrial genes (45–47). In the same manner, specific overexpression of PRDM16 in adipose tissue is sufficient to induce browning of visceral adipose tissue (48). Furthermore, beside phosphorylation events that tightly control the activity of transcription factors and co-regulators, NAD^+^-dependent deacetylase sirtuin-1 deacetylates PPARγ on two lysine residues, enabling PRDM16 recruitment and the subsequent activation of UCP1, in a rosiglitazone-dependent manner (49). It should be noted that none of these molecular regulators are specific to brown adipocytes and UCP1 expression. In particular, PPARγ, which appears crucial for the expression of UCP1, also has the great disadvantage of promoting white adipocyte differentiation (50).

### Adipose Tissue Plasticity: From Brown to Brite

As early as 1984, Young et al. described the presence of UCP1-expressing adipocytes in murine parametrial fat (1). This observation was more precisely described in later studies by Loncar (2) and Cousin et al. (3). Such apparent tissue plasticity was found to be strongly promoted after cold exposure or β3-adrenergic agonist, including in dogs (4). The relative numbers of these cells varies according to the fat pad. Subcutaneous WAT is more likely to develop UCP1-expressing cells than other depots such as mesenteric and perigonadic WAT. We therefore proposed a gradient in the plasticity of the different white fat pads similar to that described for skeletal muscles, which are classified as glycolytic, oxidative, and mixed fiber (3). Two non-exclusive hypotheses could explain the emergence of UCP1-expressing adipocytes in WAT: *de novo* differentiation from progenitors and/or transdifferentiation of mature white into brown adipocytes (51). The hypothesis of transdifferentiation is supported by several findings demonstrating that there is neither an increase in DNA amount nor a process of preadipocyte proliferation during acute cold exposure in the peri-ovarian adipose tissue (52).

It is noteworthy that, for decades, UCP1-expressing cells in white fat were considered as comparable to the classical brown adipocytes present in a typical BAT depot, i.e., the interscapular BAT. However, recent findings have strongly challenged this view and demonstrated that these cells have distinct gene expression profiles (53, 54) and different developmental origins (55–58). UCP1-expressing adipocytes in white fat thus constitute a third type of adipocytes, which have recently been named beige or brite (brown in white) adipocytes (43, 54, 59, 60). The development of these cells in WAT has been termed the browning process.

## A New Look on WAT Heterogeneity and Plasticity: Adipocytes of all Colors and Origins

It was long thought that white and brown adipocytes had a common progenitor but recent works have challenged this idea. A first step came with the proposal that classical brown adipocytes, and not brite adipocytes, derived from the paraxial mesoderm, particularly the dermomyotome, and that they originated from a precursor expressing myogenic transcription factor-5 (Myf5) (55). The commitment of the bipotent progenitor to the brown phenotype is controlled by members of the TGF-β superfamily, such as BMP7 and the PRDM16-C/EBPβ transcriptional complex, which induce the expression of PPARγ and PGC-1α (41, 61, 62). The common developmental origin of muscle cells and typical brown adipocytes is also supported by their very similar transcriptional signature (53, 54) and the old observation of the presence of glycogen stocks in brown adipocytes (63, 64). Furthermore, the demonstration that human skeletal muscle contains CD34 positive progenitor cells that can easily differentiate into brown adipocytes is another argument in favor of the link between brown adipocytes and skeletal muscle cells (65). The common origin of both types of cells could also be related to their metabolic activities, which are very close from a bioenergetics point of view. Shivering thermogenesis in muscle corresponds to the implementation of a futile cycle with a high rate of ATP synthesis associated with a high rate of ATP consumption by contraction processes. The resulting high oxidative catabolism dissipates a large amount of energy as heat. In this case, the functioning of ATP synthase resembles that of a mitochondrial proton channel such as UCP1. From a somewhat provocative point of view, brown adipocytes could be presented more as specialized muscle cells than as specialized adipocytes. Whatever the fate of such a hypothesis, this rather simplistic portrayal was quickly complicated and the developmental origin of the different types of adipocytes does not appear to be as simple as initially proposed. While brown adipocytes present in the interscapular BAT are thought to derive from Myf5-positive progenitors, those present in the brown perirenal and peri-aortic fat pads would originate from a Myf5-negative progenitor (56). Furthermore, some white and brite adipocytes could also emerge from Myf5-positive cells, according to the nature of fat deposits (56, 58). To increase the complexity further, it has been proposed that a subpopulation of brite adipocytes derives from smooth muscle cell (66).

Like the developmental origin, the cell mechanism(s) at the origin of the browning process in adults are not yet clear and are still widely debated. Consistently with the transdifferentiation hypothesis, a recent study using a transgenic mouse model with permanent or temporary staining of UCP1-expressing cells, has shown that some “phenotypically white” adipocytes can acquire a brite phenotype (i.e., displaying multilocular droplets together with UCP1 expression) after cold exposure (67). Conversely, when these mice were returned to 21°C, the brite adipocytes returned to a white phenotype (i.e., unilocular droplet), reacquiring a brite phenotype after re-exposure to cold (67). In apparent opposition to this conclusion, another study has recently demonstrated that beige adipocytes arise from *de novo* differentiation of precursors (68). The browning mechanism would also rely on different cell mechanisms according to the nature of the fat pad. While UCP1-expressing adipocytes within subcutaneous WAT would arise from the transdifferentiation phenomenon, those from the epididymal fat pad would originate from the proliferation and the differentiation of progenitor cells (69, 70). Adding to this complexity, it should not be forgotten that non-stimulated brite adipocytes could have a phenotype very close to that of the white adipocyte. This phenotype homology could lead to a misinterpretation of browning as a white adipocyte transdifferentiation when it could simply correspond to the activation of inactive brite adipocytes. It is noteworthy that, whatever the intensity of the stimuli, some unilocular white cells are totally refractory to cell plasticity. This would also mean that different types of unilocular cells lacking UCP1 expression could co-exist inside the same fat pad.

The compilation of these findings shows the current confusion, which is probably due to the underestimated complexity of the system. It seems that there are several pathways and developmental origins that give rise to unilocular UCP1 negative adipocyte(s) and to multilocular UCP1 positive adipocyte(s), without anyone knowing if cells having a similar apparent phenotype have the same functions and carry out the same regulations. This also emphasizes the lack of relevant, robust tools to identify and distinguish the different types of adipocytes because the white adipocyte phenotype is defined by its lack of brite or brown adipocyte markers, which is clearly insufficient. Although a large number of markers specifically expressed in each type of adipocytes or precursors have been proposed from gene expression profiling of isolated white, brown, and brite adipocytes and/or from studies on whole adipose tissues, their validity is currently under investigation (71). This kind of approach does not really take account of the heterogeneity of cells inside the tissues. Even in conditions of browning such as cold exposure, brite adipocytes represent only a small proportion of the tissue cells and this can lead to a biased view grouping white and brite adipocytes together. It is therefore necessary to characterize the different adipocytes in homogeneous cell populations. Also, we cannot exclude the possibility that typical brown adipocytes could co-exist with brite adipocytes in the same white fat pad since brite adipocytes can co-exist with typical brown adipocytes in typical brown fat pad.

## Unexpected Browning Inducers Reveal Putative New Roles for UCP1-Expressing Adipocytes

**Figure 1:**
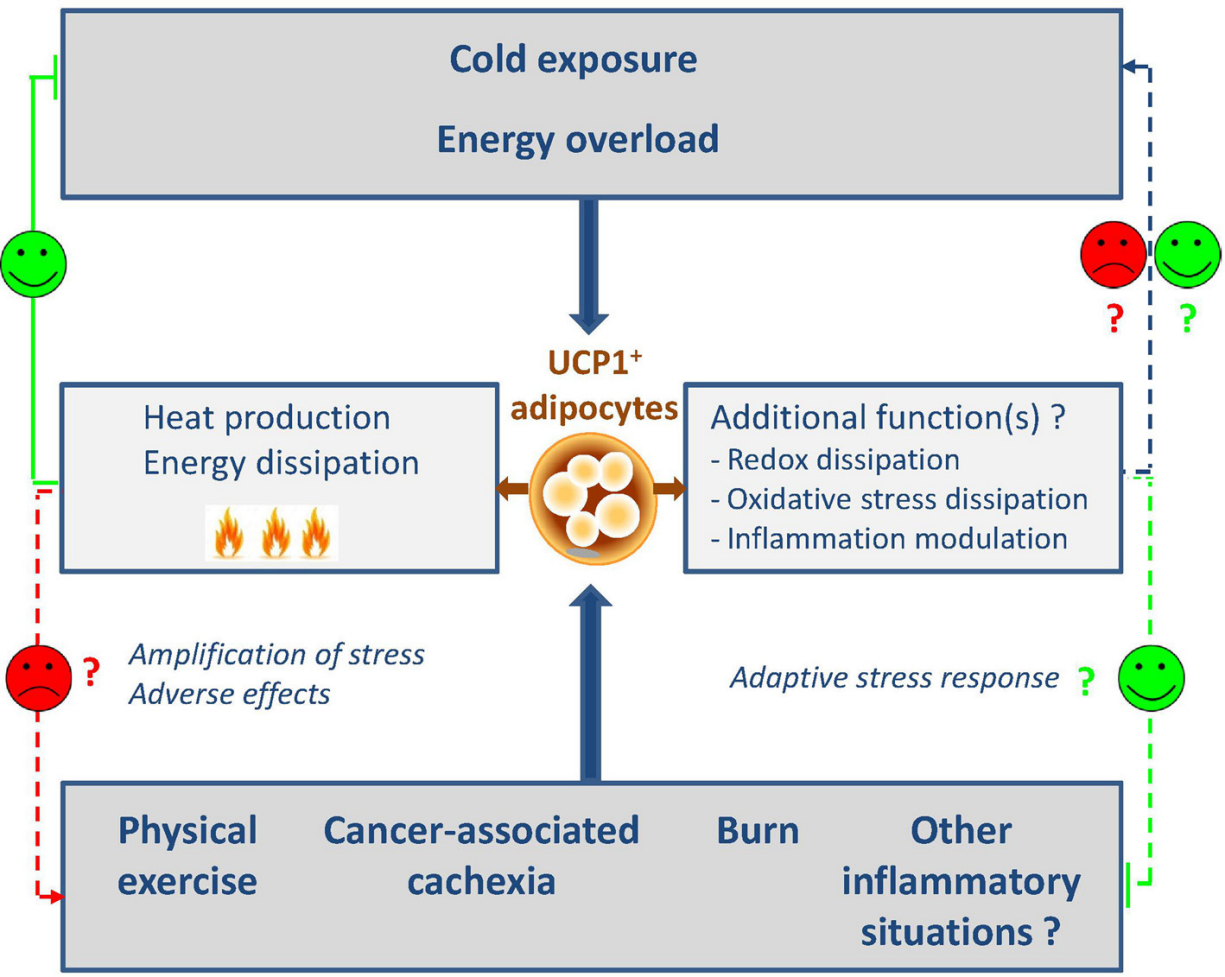
**Classic and new regulators of browning**. The induction of browning is classically described after cold exposure or high-fat diet and leads to the generation of heat and energy dissipation. In this context, browning represents a relevant target to fight against metabolic diseases. More recently, unexpected physiological or physio-pathological situations were also described as new browning inducers. These findings shed the light on putative and unexpected roles of browning such as redox and oxidative stress dissipation and inflammation modulation. On the other hand, the automatic association with heat and energy dissipation could appear as adverse side effects that will be necessary to control. The design of browning antagonists will be then relevant.

After the old failure of β3-agonist treatment in humans, the discovery of functional brown fat in adult humans has revived the research. Besides the adrenergic pathway that represents the ancestral and best described activator of UCP1 expression and in addition to thyroid hormones, several peptides have recently been identified as browning inducers or inhibitors that correspond to as many therapeutic targets. Several recent reviews summarize the different regulators of the browning process (41, 72–74). We decided to focus on the unexpected physiological or physio-pathological conditions recently found to regulate the browning process and that highlight putative new functions for these cells (Figure 1).

### Browning as an Adaptive Element in Redox Metabolism?

**Figure 2:**
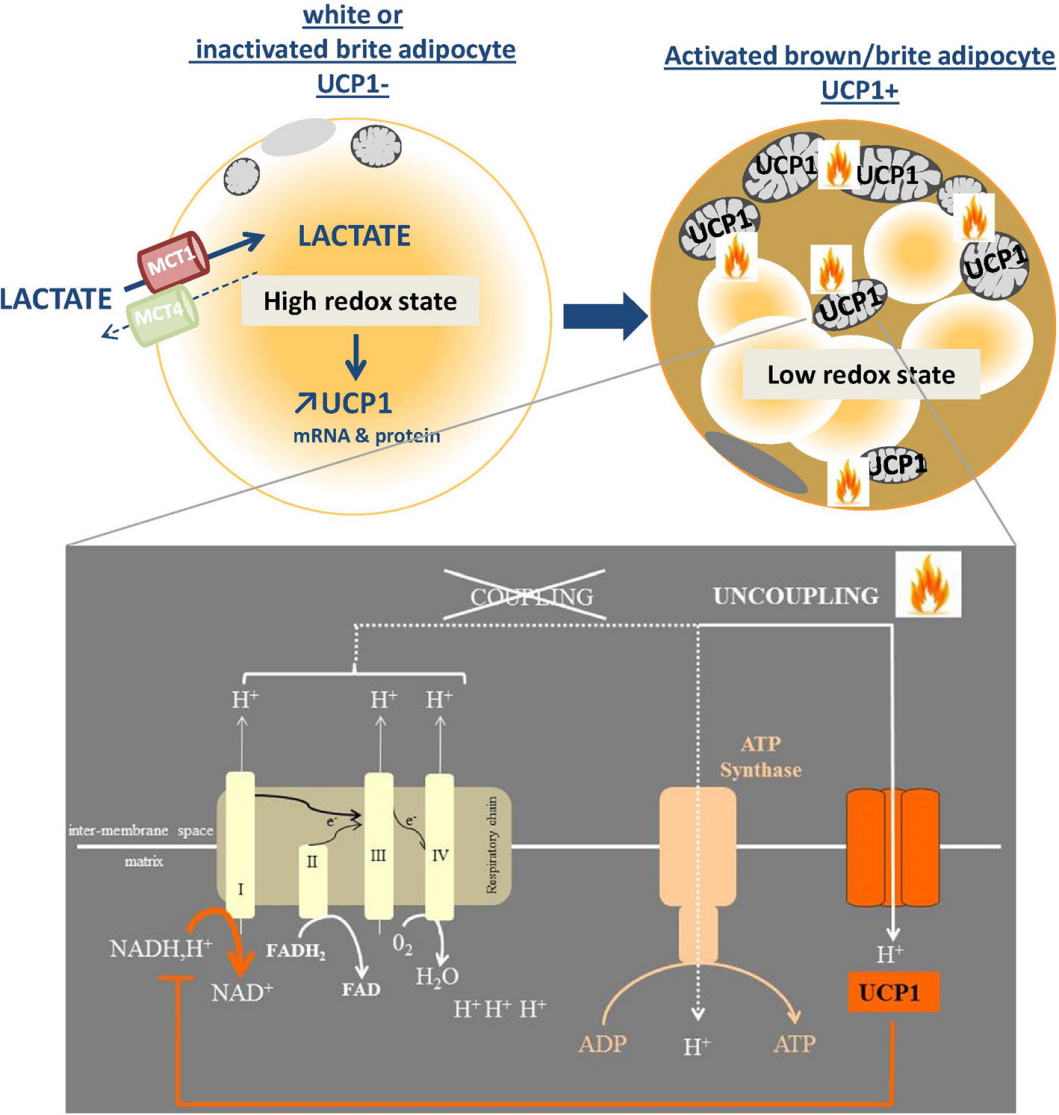
**Browning as a redox responsive mechanism**. The import of lactate via the monocarboxylate transporter MCT1 is associated with a redox state (NADH/NAD) increase which, in turn, triggers the expression of browning genes including UCP1. The effect of activated UCP1, which is inserted into the inner mitochondrial membrane, is to uncouple the respiratory chain function from the ATP synthesis. The subsequent increase in oxidative metabolism generates heat and oxidizes reduced equivalents such as NADH, which was at the origin of the up-regulated UCP1 expression.

While an increasing number of agents have been reported to stimulate browning as discussed, very few studies have investigated the role of the metabolic environment in the phenomenon. We recently demonstrated the unexpected role of redox metabolites, such as lactate, in the browning process. Lactate, which is a major product of anaerobic metabolism, promotes WAT browning through its transport by the monocarboxylate transporters (75). Lactate-induced browning is mediated by a change in the intracellular redox state (Figure 2). The transformation of lactate into pyruvate by lactate dehydrogenase B induces a higher NADH/NAD^+^ ratio and modulation of this ratio by different strategies modulates the expression of UCP1 (75). As, in turn, uncoupling the ATP production from the respiratory chain by high levels of UCP1 accelerates the oxidation of NADH by the complex I, we postulate that browning constitutes an adaptive mechanism to alleviate a strong redox pressure (75) (Figure 2). The increased expression of the lactate-importing transporter during activation of UCP1-expressing adipocytes, e.g., after cold exposure (75) suggests that lactate could also be used as a substrate by these cells. We postulate that, in the same way as for glucose and triglyceride homeostasis, UCP1-expressing cells could regulate plasma lactate levels and lactate metabolism. We propose that there is a redox regulatory loop where lactate induces a metabolic reprograming of adipocytes toward an uncoupled oxidative phenotype that, in turn, favors lactate consumption and dissipation.

This points to a totally new function for brite adipocytes and extends the role of lactate as a signaling molecule in the browning field. This signaling pathway could also be involved in the emergence of UCP1-expressing adipocytes in white fat during obesity. WAT is an important producer of lactate in physiological conditions (76, 77). It could be postulated that, in an obesity context where large adipocytes are hypoxic, they could locally produce large amounts of lactate that would stimulate the browning of neighboring white adipocytes and/or the recruitment of brite progenitors that promote energy dissipation (77). This link between browning and redox state is also emphasized by two recent studies, demonstrating that UCP1 expression is strongly controlled by different redox elements such as oxidative stress and antioxidant defenses. Sestrins, stress-inducible proteins that limit oxidative stress, inhibit UCP1 expression (78) while glutathione deficit, one of the key elements of the cytosolic antioxidant defenses, increases it (79). Oxidative stress and redox state thus constitute new and important regulators of UCP1 expression.

### Browning as a Non-Specific Stress Response?

Three unexpected physiological or pathophysiological situations were found to be associated with browning: exercise, cancer-associated cachexia, and burns. Before our findings, physical exercise had been shown to activate BAT and WAT browning (80, 81). In addition to IL-6 (82), two muscle-derived hormones, irisin and meteorin-like, are believed to mediate such effects (80, 83). The expression and the impact of irisin in humans have been strongly debated but its existence has been unequivocally demonstrated by a recent study using tandem mass spectrometry (84). Irisin is detectable in the blood of untrained persons during exercise or exposure to low temperatures (84, 85). While some studies have shown that irisin has no effect on the differentiation of human preadipocytes toward the brite phenotype (86), others have demonstrated that irisin activates the thermogenic activity of preadipocytes extracted from human neck (87). Additional signals, including lactate, whose plasma levels rise strongly during physical activity, could also contribute to exercise-induced UCP1 expression (75). The second situation, cachexia, is related to physiopathology associated with wasting syndromes, such as diabetes, sepsis, and cancer (88). It results in chronic inflammation and severe weight loss associated with atrophy of fat deposits and muscles. It is often associated with high plasma levels of lactate and usually precedes the death of the patient (89). Several studies have demonstrated that cachexia induced by the injection of cancer cells is associated with browning of WAT and an increase in energy expenditure (90–92). Inflammation and, especially, the production of interleukin-6 play a key role in this phenomenon and the lack of interleukin-6 receptor blocks browning induced by cancer cells (90). Parathyroid hormone-related protein may also mediate cancer-cell-induced browning of WAT (91). Lastly, in addition to exercise and cachexia, severe burns are associated with hypermetabolic response, hyperthermia, and browning in rodents and in humans (93, 94).

The role(s) of UCP1-expressing cells in these different conditions still remain to be unraveled. The fact that muscle contraction during exercise promotes the emergence and activation of energy-dissipating and thermogenic cells does not seem physiologically consistent with the energy conservation theory. This discrepancy would be explained if muscle-derived signals that induce browning could be shown to have evolved from shivering-related muscle contraction (87). In this scenario, WAT browning would represent a coherent and additive mechanism in thermoregulation promoted by shivering. For cachexia, associated browning could constitute an adaptive thermoregulatory response to fight against the hypothermia associated with the decrease of both fat insulation and heat-related muscle mass observed during cachexia. Addressing this issue would require these results to be reproduced at thermoneutrality, as illustrated by the study conducted by Champigny et al. in starving conditions (95). In this study, the authors found an up-regulation of UCP1 expression and brown adipocyte activation during starvation, which are physiologically inconsistent (95). In fact, starvation-induced UCP1 expression disappeared when mice were kept at thermoneutrality, suggesting that UCP1 up-regulation was an adaptive thermogenic response of rats to fight against the temperature deficit associated with starvation-induced torpor (95). Browning is always associated with thermoregulation responses in these situations but could have adverse metabolic effects that need to be controlled. For massive burn injury, browning would be triggered by the large stress release of catecholamine and inflammatory signals and would participate in the increase of the whole body metabolic rate (94). In fact, the role of inflammatory signals and cytokines on the browning process was first reported during cold exposure (96). Cyclooxygenase-2, whose expression is increased in WAT during cold exposure, is thought to trigger the browning process through the synthesis of prostaglandins E2 and I2 (97, 98). The link between prostaglandins and UCP1 is not new and it has been proposed that the natural ligand of PPARγ is prostaglandin PGJ2 (99). However, these results have been questioned following the recent publication of the opposite effect of prostaglandins E2 and F2α on the expression of UCP1 (100). Surprisingly, immune cells are involved in cold-induced browning as eosinophils express and secrete interleukin-4 and interleukin-13, which recruit alternative M2 macrophages in the subcutaneous WAT during cold exposure (101). These macrophages secrete catecholamines that induce browning. The meteorin-like hormone secreted by both WAT and BAT during cold exposure or during physical exercise has the same effect on eosinophils (83). At the same time, another group has demonstrated that the activation by interleukin-33 of Group 2 innate lymphoid cells (ILC2s), known for their role in tissue remodeling, could also induce the recruitment of eosinophils via the release of interleukin-13 (102). ILC2s may also, in response to interleukin-33, produce and secrete methionine-enkephalin, a new browning inducer (103). Although all these mechanisms were discovered in the context of cold exposure, it is reasonable to speculate that any events able to activate ILC2s or eosinophils are able to promote browning. Together with the fact that interferon regulatory factor-4 controls UCP1 expression (104), these observations bring to light an undiscovered link between browning and inflammation/immunity.

The consequences of the apparition of UCP1-expressing cells in such inflammation- and injury-related scenarios currently remain poorly understood but it is noteworthy that the common point between all the situations is the notion of intense stress and inflammation. For this reason, with the limits concerning thermoregulation discussed previously and although these fields are evolving rapidly, we can already assume that browning could appear in any severe stress conditions. Whether these cells have positive or negative effects on such stress and loss of tissue homeostasis conditions is unknown (Figure 1). They could have adverse effects through increased energy dissipation in wasting syndromes and injury conditions but also could play positive roles through unexpected functions such as redox and oxidative stress dissipation, metabolite consumption, or even anti-inflammatory actions. This is an underexplored scientific area that will remain very open for the coming years.

## Perspectives in Humans

In humans, it has long been considered that BAT is only present in neonates, with some exceptions such as patients suffering from pheochromocytoma (105, 106) or workers exposed to cold temperatures (107). Mainly localized in the interscapular region like the main murine BAT depot, the BAT present in new-born babies enables heat to be produced to combat the thermal stress associated with birth. While this fat pad disappears rapidly and is absent in human adults like in adult sheep (13), some histological studies performed in the 1970s claimed that human adults possessed some brown fat deposits in additional zones, including around the kidney, heart, and adrenal gland, and along the aorta and carotid (108). However, these studies were unable to change the general opinion that human adults did not possess BAT. Recently, this view has been contradicted by medical imaging based on the measurement of glucose uptake (using 18-fluorodesoxyglucose associated with positron emission tomography–computed tomography) This approach, widely used in oncology to detect tumors, when associated with histological and immunocytochemistry studies, highlighted the presence of BAT in healthy human adults (109–112). The activity of BAT is inversely correlated with body mass index, suggesting that it could play a role in energy homeostasis (109–112). Given their high oxidative potential and their ability to consume nutrients such as glucose and triglycerides, brown adipocytes in humans possess very attractive properties for fighting against metabolic diseases, including type II diabetes and obesity (30). While molecular profiling studies have shown that human brown adipocytes are very similar to brite adipocytes described in mice (59, 113, 114), some others claim that brown fat deposits in humans are made of a mixture of brown and brite adipocytes, the differential distributions of which appear to depend on their anatomical location, consistently with our previous remarks. In the neck region, brown adipocytes would be located deep within the tissues and brite adipocytes rather at the surface (115, 116). The search for brite progenitors in human WAT led to the discovery of bipotent (white and brite) progenitors that expressed the tissue non-specific alkaline phosphatase marker (117), while CD29 enabled the isolation of brown progenitors present in the deep regions of the human neck (118). Among the putative targets (72), we cannot ignore the β3-adrenergic pathway. Although many efforts failed to find suitable compounds (119), new pharmacological and genetic studies have revealed that targeting this pathway could be efficient (120–122). While PPARγ is a very potent inducer of UCP1 expression, it also causes many side effects, including body weight gain and cardiovascular issues, that strongly impair its therapeutic use (50). One way to bypass this issue would be to design a selective PPAR modulator (123, 124). Recently, oral supplementation of the bile acid chenodeoxycholic acid has been shown to activate BAT and to increase whole body energy expenditure in humans, making bile acids attractive candidates for counteracting obesity and related metabolic diseases (125).

By contrast, recent works on inflammation and burns have also revealed the bad and the ugly of browning as previously described. While it is important to find new browning inducers to limit energy stores and metabolic diseases, it also appears crucial to develop new browning inhibitors to limit adverse energy dissipation in wasting-associated syndromes.

## Conclusion

All these recent discoveries constitute a very active field attracting groups of scientists who are expert not only in metabolism but also in immunology, development, cancer, etc. This has shed new and unexpected light on brown and brite adipocytes and has already given original insights into the putative role of these cells. For the future, it is reasonable to speculate that the story is not finished because this field is now wide open and raises new questions and hypotheses, keeping in mind the key ancestral role of UCP1 in thermogenesis and bioenergetics.

## Conflict of Interest Statement

The authors declare that the research was conducted in the absence of any commercial or financial relationships that could be construed as a potential conflict of interest.
